# Normative reference ranges for echocardiographic chamber dimensions in a healthy Central European population: results from the Czech post-MONICA survey

**DOI:** 10.1186/s12947-019-0172-0

**Published:** 2019-10-30

**Authors:** Josef Marek, Jean-Claude Lubanda, Renata Cifkova, Petr Kuchynka, Lubor Golan, Eduard Nemcek, Ales Linhart

**Affiliations:** 1 2nd Department of Medicine – Department of Cardiovascular Medicine, First Faculty of Medicine, Charles University and General University Hospital in Prague, U Nemocnice 2, 128 08 Praha 2, Czech Republic; 2 Center for Cardiovascular Prevention, First Faculty of Medicine, Charles University in Prague and Thomayer Hospital, Prague, Czech Republic

**Keywords:** Echocardiography, Left ventricular function, Left ventricular mass, Left ventricular end-diastolic dimension, Right ventricular function, Atrium

## Abstract

**Background:**

Normative reference values for echocardiographic chamber quantification are of great importance; however, this can be challenging. Our aim was to derive these values including degrees of abnormality from a random Central European population sample with a homogeneous subset of healthy subjects.

**Methods:**

We analysed echocardiograms obtained in a randomly selected population sample during the Czech post-MONICA survey in 2007/2008. Overall, 1850 out of 2273 persons of the whole sample of three districts had adequate echocardiograms (81.4%). A healthy subgroup defined by the absence of known cardiovascular disease was used to define normal reference range limits (*n* = 575, median age 42 years [IQR 34–52], 57% females). The whole population sample with predefined percentile cut-offs was used to define degrees of abnormality.

**Results:**

Left ventricular (LV) size tended to decrease with age, while LV mass increased with age in both males and females and in both the healthy and general populations. LV dimensions were larger in males, except for body surface area-indexed LV diameter. M-mode derived LV measurements were larger and LV mass higher compared to 2D measurements. Right ventricle basal dimension was larger in males.

**Conclusions:**

Our study provides reference ranges for echocardiographic measurements obtained in a healthy subgroup derived from an epidemiological study of a Central European population. Where feasible, degrees of abnormality are provided based on the whole population sample including patients with disease. Our data show that age, gender and measurement method significantly affect cardiac dimensions and function and should be always taken into account.

## Introduction

Echocardiography is the most commonly used imaging method to evaluate cardiac structure and function [1]. Quantification of cardiac chamber dimensions and function remains paramount to echocardiographic examination, defining normal values is thus of great importance. However, this can be challenging due to variations based on gender, age and specific populations. Indeed, recent data show substantial influences of ethnicity, gender and age, while only limited epidemiological data exist uniquely for Central European populations [2, 3]. Furthermore, with a healthy population, only normal reference limits can be reliably calculated. For defining degrees of abnormality, a true general population sample is advantageous [4, 5]. Therefore, we aimed to define normal chamber dimensions and function based on a randomly selected population sample in the Czech Republic.

## Methods

### Study population

The Czech post-MONICA study is a population-based survey assessing cardiovascular risk profile in a randomly selected sample of the Czech population. Detailed methods of the study have been described previously [6, 7]. Briefly, one-percent adult population samples stratified by age and gender were randomly selected from the general population of nine districts of the country. Selection was made using the General Health Insurance Company registry that keeps, by law, a list of people who are insured. Since health insurance is mandatory for Czech citizens, the registry covers the entire population. Echocardiographic examination was performed in three districts (Benesov, Pardubice, and city of Pilsen). The present analysis includes 1850 individuals (i.e. 81.4% of the entire screened population, *n* = 2273, within the three districts in 2007/2008) over 25 years of age in whom echocardiograms were available and these scans were used for subsequent analysis. The study was approved by the joint ethics committee of the Institute for Clinical and Experimental Medicine and Thomayer Hospital and was in accordance with the Declaration of Helsinki.

**Fig. 1:**
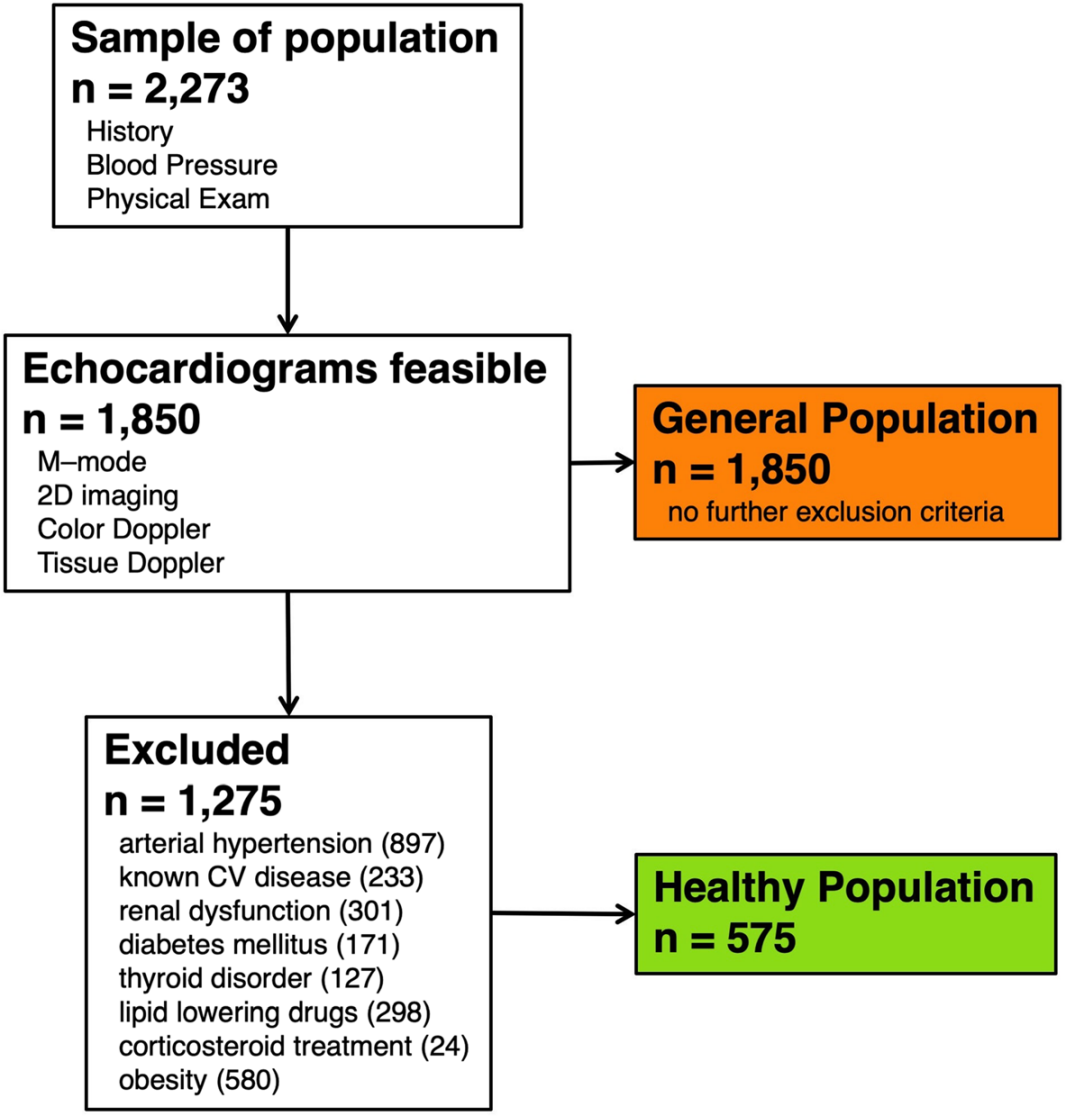
Flowchart showing selection of patients for the present study. The population was randomly selected from the country’s three districts as part of the Czech post-MONICA study 2007–2008. CV, Cardiovascular. The numbers in parentheses indicate amount of patients that were excluded for that specific reason

As Fig. 1 shows, the healthy cohort was defined as a subset of the general sample without obesity (BMI >  30 kg/m^2^), arterial hypertension, known cardiovascular disease, renal dysfunction, diabetes mellitus, thyroid disorder, lipid-lowering or corticosteroid treatment. The healthy cohort included 575 selected patients. The total cohort consisted of all the recruited individuals irrespective of treatment or comorbidities.

### Echocardiography

All studies were done using the GE Vivid 7 system (GE Healthcare, Chicago, Illinois, USA). Three measurements were taken for each parameter and averaged. All measurements were done according to the recommendations for ASE/EACVI chamber quantification unless otherwise specified [4] and were done in one centralized core laboratory. Briefly, aortic root and left atrial (LA) diameters were measured from the parasternal long-axis view using M-mode and both measurements were performed using the leading edge to leading edge convention.

Left ventricular (LV) dimensions were measured first using 2D guided M-mode acquisition, with diameters measured according to the ASE convention [1]. The interventricular septum and posterior wall thickness and LV diastolic diameter were also measured with the 2D technique using the blood-myocardial interface. LV mass was calculated using the modified Devereux formula, from both M-mode and 2D recordings as previously described [8].

LV volumes and ejection fraction were measured from the single plane apical four-chamber view using the Simpson rule. Left atrium (LA) volume was measured using the area-length method while vertical and horizontal dimensions of both atria were measured as perpendicular major and minor axes.

Right ventricular (RV) basal dimension was measured as the largest diameter in the basal third of the right ventricle in end-diastole. Tricuspid annular plane systolic excursion (TAPSE) was measured from dedicated M-mode recordings.

Tissue Doppler recording was made on the septal and lateral sides of the mitral annulus and on the tricuspid annulus using dedicated pulsed wave tissue Doppler acquisitions. Peak systolic velocity was denoted s’.

### Statistical analysis

**Fig. 2:**
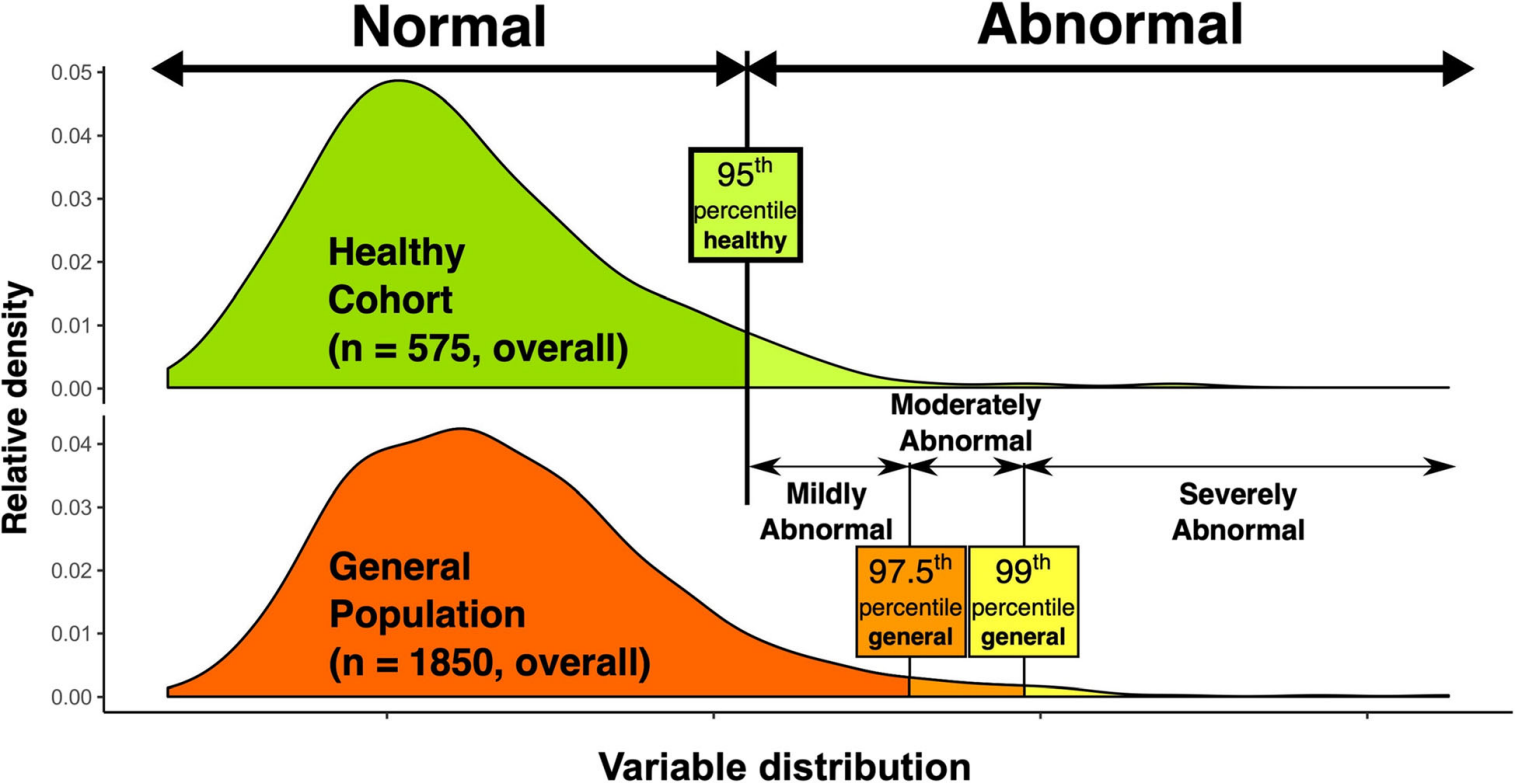
Illustration showing definition of reference ranges. Upper part of the figure shows distribution in healthy individuals, with the 95th quantile used to define abnormal value. Lower part of the figure shows the ways for defining different degrees of abnormality. Patients falling above the 95th percentile of the healthy population but under the 97.5th percentile of the general population were classified as mildly abnormal; those between the 97.5th and 99th percentiles of the general population as moderately abnormal and patients over the 99th percentile of the general population as severely abnormal. The actual distribution density of this illustration was based on LV atrial volume distribution in males (indexed to BSA). BSA, body surface area; LV, left ventricle

Continuous values are summarized using median with 25th to 75th percentile and categorical variables using proportions. The Mann-Whitney U test and Chi-square test were used to compare baseline variables and Wilcoxon signed-rank test to compare 2D and M-mode measurements. Reference values for normality and degrees of abnormality were calculated using multivariate quantile regression with age and gender as predictors. Limit of normality was defined as 5th or 95th percentile of the respective value in the healthy subset. Gender specific cut-offs are provided for all variables and age-specific cut-offs are presented in variables where the testing showed relevant age differences. We stratified degrees of abnormality using the whole population sample including both healthy and remaining patients. Cut-offs for moderate abnormality were 2.5th or 97.5th interval of the *whole* sample and severe abnormality as 1st or 99th percentile of the *whole* sample. These differences are summarized in Fig. 2. Nonparametric methods with quantile regression were preferred because of non-normal distribution of echocardiographic variables (Shapiro-Wilks test *p* <  0.001 for all echocardiographic variables for both healthy and general populations). Using cut-offs based on distribution of general population has been suggested previously [4, 5]. A *p* value < 0.05 was considered significant. Analysis was done using R software version 3.0.2 (R Foundation for Statistical Computing, Vienna, Austria).

## Results

### Baseline variables

Overall, as Table 1 shows, the healthy cohort was on average younger, with a higher percentage of females and lower body weight, height, body surface area (BSA), body mass index (BMI), systolic and diastolic blood pressure. Healthy patients also had lower total cholesterol and triglycerides and higher HDL cholesterol and showed a numeric trend towards lower LDL levels. All patients in both subsets were Caucasians. The distribution of BSA, height and weight according to age groups is shown in Additional file 2: Table S9.

**Table 1:** Baseline characteristics

Variable	Healthy subset *n* = 575	Remaining cohort *n* = 1275	*p* value
Age (years)	42 [34, 52]	58 [49, 65]	< 0.001
Female gender, n (%)	328 (57%)	631 (49%)	0.003
BSA (m^2^)	1.82 [1.70, 1.99]	1.97 [1.82, 2.13]	< 0.001
Weight (kg)	72 [62, 81]	86 [74, 98]	< 0.001
Height (cm)	171 [165, 179]	171 [163, 177]	0.003
BMI (kg/m^2^)	24 [22, 26]	29 [26, 33]	< 0.001
Systolic blood pressure (mmHg)	118 [109, 124]	132 [121, 144]	< 0.001
Diastolic blood pressure (mmHg)	78 [72, 81]	83 [78, 90]	< 0.001
History of CVD, n (%)		230 (18%)	
Antihypertensive medication, n (%)		606 (48%)	
Diabetes mellitus, n (%)		171 (13%)	
Lipid-lowering drugs, n (%)		298 (23%)	
Total serum cholesterol (mmol/l)	5.02 [4.42, 5.71]	5.25 [4.57, 5.92]	< 0.001
Total serum triglycerides (mmol/l)	0.97 [0.73, 1.39]	1.39 [1.03, 2.02]	< 0.001
HDL cholesterol (mmol/l)	1.51 [1.27, 1.76]	1.34 [1.10, 1.62]	< 0.001
LDL cholesterol (mmol/l)	2.94 [2.37, 3.58]	3.05 [2.41, 3.65]	0.091
Age group (n)			< 0.001
under 40 years	239	160	
40–60 years	283	556	
over 60 years	53	559	

Continuous variables presented as median [25th, 75th percentile]

*BMI* body mass index, *BSA* body surface area, *CVD* cardiovascular disease

### Effects of age, gender and measurement method

Tables 2 and 3 show the effects of gender and age on reference limits (the 5th or 95th percentile of healthy population). Values stratified by age and gender as well as effect of age and gender on median values are shown in Additional file 1: Tables S5 and S6, Additional file 2: Tables S7 and S8. Overall, there was a slight trend towards smaller LV size upper reference limits with increasing age. LV diameter upper reference limits decreased with age when measured using the 2D method. End-diastolic and end-systolic volume upper reference limit overall decreased with age. Longitudinal LV systolic function lower reference limit measured by s’ significantly decreased. There was a strong tendency of LV mass and LV wall thickness upper reference limit to increase with age in the healthy cohort both using M-mode and 2D calculations and different indexations. There seemed to be an overall numeric trend towards enlargement of LA upper reference limit with age, significant in indexed LA diameter. Indexed right atrial vertical dimension and indexed aortic root diameter upper reference limits increased significantly with age.

**Table 2:** Effects of age and gender on reference limits – left ventricle

Variable	Age effect (per 10 years)	Age effect *p* value	Gender effect (male vs. female)	Gender effect *p* value
Left ventricular dimensions				
2D method				
LV end-diastolic diameter (mm)	−0.7 [−1.0, − 0.3]	0.001	5.6 [4.3, 7.0]	< 0.001
LV end-diastolic diameter, BSA (mm/m^2^)	−0.4 [− 0.8, − 0.1]	0.021	− 1.4 [− 2.5, − 0.4]	0.007
M-mode method				
LV end-diastolic diameter (mm)	0.5 [− 0.1, 1.2]	0.113	5.4 [4.0, 6.8]	< 0.001
LV end-diastolic diameter, BSA (mm/m^2^)	0.3 [−0.1, 0.7]	0.107	−1.3 [−2.1, − 0.4]	0.004
LV end-systolic diameter (mm)	0.7 [− 0.2, 1.6]	0.114	4.1 [1.9, 6.2]	< 0.001
LV end-systolic diameter, BSA (mm/m^2^)	0.4 [−0.1, 0.9]	0.109	−0.4 [−1.5, 0.7]	0.469
LV mass and wall thickness				
2D method				
Interventricular septum (mm)	0.5 [0.1, 0.8]	0.011	1.3 [0.4, 2.3]	0.004
LV Posterior wall (mm)	0.4 [0.2, 0.6]	< 0.001	1.5 [1.0, 2.0]	< 0.001
LV mass, BSA (g/m^2^)	4.2 [2.1, 6.4]	< 0.001	23.5 [18.4, 28.6]	< 0.001
M-mode method				
Interventricular septum (mm)	0.6 [0.3, 0.9]	< 0.001	1.7 [0.9, 2.5]	< 0.001
LV Posterior wall (mm)	0.6 [0.4, 0.8]	< 0.001	1.6 [1.1, 2.0]	< 0.001
LV mass, BSA (g/m^2^)	7.1 [3.2, 11.1]	< 0.001	22.6 [10.6, 34.5]	< 0.001
LV mass, height^2.7^ (g/m)	4.2 [2.2, 6.2]	< 0.001	4.8 [−0.2, 9.8]	0.060
LV volumes and function				
LV end-diastolic volume (ml)	−6.6 [− 10.0, −3.1]	< 0.001	35.4 [25.1, 45.6]	< 0.001
LV end-diastolic volume, BSA (ml/m^2^)	−3.1 [− 6.5, 0.4]	0.081	8.4 [0.9, 16.0]	0.029
LV end-systolic volume (ml)	−3.6 [−5.0, −2.1]	< 0.001	23.2 [19.4, 27.0]	< 0.001
LV end-systolic volume, BSA (ml/m^2^)	−2.1 [−3.5, − 0.8]	0.002	6.4 [2.9, 9.9]	< 0.001
LV ejection fraction (%)	1.4 [− 0.1, 2.9]	0.059	− 1.6 [−5.7, 2.6]	0.455
Mitral septal s’ (cm/s)	− 0.2 [− 0.4, − 0.1]	0.001	−0.1 [− 0.4, 0.3]	0.658
Mitral lateral s’ (cm/s)	− 0.3 [− 0.5, − 0.1]	0.002	0.4 [− 0.1, 0.8]	0.162

Values depict effect size on reference limit (95th or 5th percentile of the healthy subset) with 95% confidence interval

*BSA* body surface area, *LV* left ventricle

**Table 3:** Effects of age and gender on reference limits – left atrium, right chambers, aorta

Variable	Age effect (per 10 years)	Age effect *p* value	Gender effect (male vs. female)	Gender effect *p* value
Left atrium				
LA diameter M-mode (mm)	0.7 [− 0.2, 1.7]	0.138	4.3 [2.1, 6.6]	< 0.001
LA diameter M-mode, BSA (mm/m^2^)	1.0 [0.5, 1.4]	< 0.001	− 1.3 [− 2.2, − 0.3]	0.009
LA vertical diameter (mm)	−0.2 [− 1.4, 1.0]	0.752	4.2 [0.2, 8.2]	0.042
LA horizontal diameter (mm)	1.1 [−0.0, 2.3]	0.056	1.6 [−1.0, 4.2]	0.232
LA volume (ml)	1.1 [−2.7, 5.0]	0.571	15.4 [4.0, 26.9]	0.008
LA volume, BSA (ml/m^2^)	1.6 [−0.3, 3.5]	0.097	0.2 [−4.8, 5.3]	0.924
Right ventricle				
RV basal diameter (mm)	0.9 [−0.2, 1.9]	0.109	6.9 [3.7, 10.0]	< 0.001
RV basal diameter, BSA (mm/m^2^)	0.4 [−0.0, 0.9]	0.066	0.1 [−1.0, 1.2]	0.839
Tricuspid s’ (m/s)	−0.2 [− 0.6, 0.2]	0.261	− 0.7 [− 1.6, 0.3]	0.194
TAPSE (mm)	− 0.6 [− 1.5, 0.2]	0.144	−0.0 [−2.3, 2.2]	0.992
Right atrium				
RA vertical diameter (mm)	−0.0 [− 0.7, 0.7]	1.000	5.7 [4.1, 7.3]	< 0.001
RA horizontal diameter (mm)	−0.5 [−1.4, 0.3]	0.202	6.3 [4.3, 8.3]	< 0.001
RA vertical diameter, BSA (mm/m^2^)	0.6 [0.1, 1.0]	0.009	−1.6 [−2.4, −0.8]	< 0.001
RA horizontal diameter, BSA (mm/m^2^)	0.1 [−0.4, 0.6]	0.663	−0.5 [−1.8, 0.8]	0.443
Aorta				
Aortic root (mm)	0.8 [−0.1, 1.8]	0.084	5.5 [3.4, 7.7]	< 0.001
Aortic root, BSA (mm/m^2^)	0.4 [0.0, 0.8]	0.045	−0.3 [−1.2, 0.7]	0.589

Values depict effect size on reference limit (95th or 5th percentile of the healthy subset) with 95% confidence interval

*BSA* body surface area, *LA* left atrium, *RA* right atrium, *RV* right ventricle, *TAPSE* tricuspid annular plane systolic excursion

LV diameter reference limits were larger in males, but this finding was completely reversed when indexed for BSA. In contrast, both end-systolic and end-diastolic LV volumes limits remained larger in males despite indexation. LV mass upper reference limit was also larger in males. Males had a significantly larger non-indexed RV basal diameter upper reference limit by over 6 mm, a trend that disappeared after BSA indexation to BSA.

**Fig. 3:**
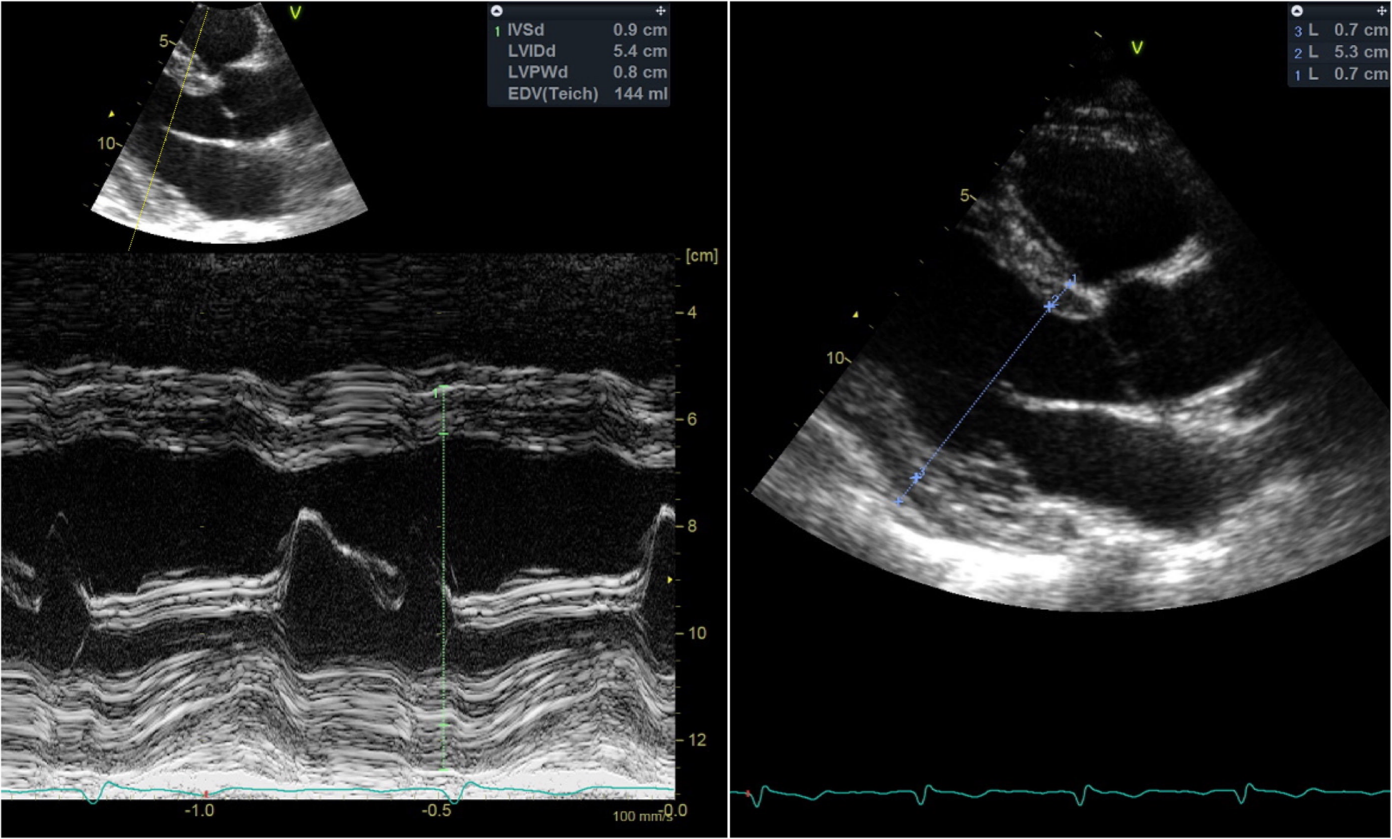
This image in parasternal long-axis view illustrates some limitations of the M-mode method of measuring LV dimension and wall thickness in some patients. In the M-mode image on the left, an oblique cut leads to multiple problems. First is incorrect measurement of interventricular septum and possible confounding from right ventricular trabeculations. Furthermore, the posterior wall is difficult to measure due to many trabeculations in the basal part of the ventricle and this leads to possibly incorrect measurement. Also, the LV dimension was slightly larger with M-mode measurement. 2D imaging on the left side can achieve a proper geometric cut and slightly smaller wall thickness measurements. The yellow dotted line on the left image has been added to make the M-mode cut line more clearly visible

Overall, M-mode derived LV linear measurements were significantly larger compared to their 2D counterparts (*p* <  0.001 for all comparisons). An example of such discrepancy is shown in Fig. 3. Furthermore, BSA-indexed LV mass using M-mode was significantly higher compared to the 2D method (*p* <  0.001).

### Estimated cut-off values

General reference values for normality irrespective of age are shown in Tables 4 and 5 separated by gender. For variables significantly affected by age, we provide further age-related cut-offs in Additional file 1: Tables S1-S4. Parametric summaries including stratification by age and gender are included in Additional file 1: Tables S5 and S6, Additional file 2: Tables S7 and S8. As for LV mass and LA volume, distributions with reference limits are shown in Additional file 1: Figure S1 to give a clearer visual idea of their distribution. Furthermore, for LV mass, Additional file 1: Figure S2 summarizes differences due to age, gender, measurement technique and population subset.

**Table 4:** Reference limits for left ventricular measurements

Variable	Males			Females		
	Abnormal	Moderately abnormal	Severely abnormal	Abnormal	Moderately abnormal	Severely abnormal
LV dimensions						
2D method						
LV end-diastolic diameter (mm)	> 56	> 60	> 63	> 51	> 56	> 58
LV end-diastolic diameter, BSA (mm/m^2^)	> 28	> 30	> 31	> 30	> 31	> 32
M-mode method						
LV end-diastolic diameter (mm)	> 60	> 64	> 66	> 55	> 59	> 63
VLV end-diastolic diameter, BSA (mm/m^2^)	> 31	> 32	> 33	> 32	> 33	> 34
LV end-systolic diameter (mm)	> 40	> 44	> 49	> 36	> 39	> 41
LV end-systolic diameter, BSA (mm/m^2^)	> 20	> 21	> 23	> 21	(> 21)^a^	(> 23)^a^
LV mass and wall thickness						
2D method						
Interventricular septum (mm)	> 11.5	> 14.0	> 15.0	> 10.3	> 12.3	> 13.3
Posterior wall (mm)	> 10.7	> 12.3	> 13.0	> 9.3	> 11.0	> 11.7
LV mass, BSA (g/m^2^)	> 107	> 130	> 138	> 82	> 111	> 128
M-mode method						
Interventricular septum (mm)	> 12.7	> 14.3	> 15.0	> 11.0	> 12.7	> 13.7
Posterior wall (mm)	> 11.0	> 13.3	> 14.0	> 9.7	> 11.5	> 12.3
LV mass, BSA (g/m^2^)	> 122	> 147	> 167	> 104	> 129	> 141
LV mass, height^2.7^ (g/m)	> 53	> 68	> 76	> 47	> 69	> 76
LV volumes and function						
LV end-diastolic volume (ml)	> 150	> 181	> 205	> 109	> 136	> 157
LV end-diastolic volume, BSA (ml/m^2^)	> 73	> 85	> 94	> 63	> 73	> 85
LV end-systolic volume (ml)	> 69	> 81	> 99	> 44	> 56	> 71
LV end-systolic volume, BSA (ml/m^2^)	> 33	> 39	> 46	> 25	> 30	> 37
LV ejection fraction (%)	< 50	< 46	< 42	< 53	< 50	< 47
Mitral septal s’ (cm/s)	< 6.0	< 5.0	< 4.3	< 6.0	< 5.0	< 4.3
Mitral lateral s’ (cm/s)	< 6.3	< 5.0	< 4.3	< 6.0	< 5.3	< 5.0

Mildly abnormal is defined as >95th or < 5th percentile of the healthy subset, moderately abnormal as > 97.5th or < 2.5th percentile and severely abnormal as >99th or < 1st percentile of the whole sample

*BSA* body surface area, *LV* left ventricle

^a^Not recommended due to negligible difference between the healthy subset and general population, see Limitations

**Table 5:** Reference limits for left atrium, right chambers and aorta

Variable	Males			Females		
	Abnormal	Moderately abnormal	Severely abnormal	Abnormal	Moderately abnormal	Severely abnormal
Left atrium						
LA diameter M-mode (mm)	> 46	> 53	> 56	> 41	> 48	> 51
LA diameter M-mode, BSA (mm/m^2^)	> 23	> 25	> 27	> 24	> 26	> 27
LA vertical diameter (mm)	> 61	> 67	> 71	> 56	> 61	> 63
LA horizontal diameter (mm)	> 48	> 52	> 54	> 46	> 50	> 54
LA volume (ml)	> 86	> 108	> 123	> 70	> 90	> 105
LA volume, BSA (ml/m^2^)	> 42	> 52	> 59	> 40	> 48	> 53
Right ventricle						
RV basal diameter (mm)	> 46	> 47	> 48	> 39	> 42	> 43
RV basal diameter, BSA (mm/m^2^)	> 23	(> 23)^a^	(> 24)^a^	> 23	(> 24)^a^	(> 24)^a^
Tricuspid s’ (m/s)	< 9.0	< 8.0	< 7.0	< 9.0	< 8.0	< 7.7
TAPSE (mm)	< 19	< 17	< 15	< 19	< 18	< 17
Right atrium						
RA vertical diameter (mm)	> 57	> 61	> 65	> 51	> 56	> 58
RA horizontal diameter (mm)	> 50	(> 50)^a^	(> 53)^a^	> 42	> 45	> 47
RA vertical diameter, BSA (mm/m^2^)	> 28	> 30	> 32	> 30	> 31	> 33
RA horizontal diameter, BSA (mm/m^2^)	> 25	(> 25)^a^	(> 26)^a^	> 25	> 26	> 27
Aorta						
Aortic root (mm)	> 40	> 42	> 43	> 34	> 36	> 37
Aortic root, BSA (mm/m2)	> 19	> 20	> 21	> 20	> 21	> 22

Mildly abnormal is defined as >95th or < 5th percentile of the healthy subset, moderately abnormal as > 97.5th or < 2.5th percentile and severely abnormal as >99th or < 1st percentile of the whole sample

*BSA* body surface area, *LA* left atrium, *RA* right atrium, *RV* right ventricle, *TAPSE* tricuspid annular systolic plane excursion

^a^Not recommended due to negligible difference between the healthy subset and general population, see Limitations

## Discussion

In our study, we calculated reference limits for chamber dimensions and systolic function of the left and right ventricles using a pre-specified healthy subset from a population-wide epidemiological survey. Furthermore, data from our unselected general population sample allowed us to propose cut-offs for mild, moderate and significant abnormality, based on the approach using predefined percentiles and unselected population sample [4, 5]. The major strengths of our study are the homogeneity of the studied population, which was randomly selected and is considered representative of the Czech population, and analysis performed by skilled operators trained to use a standardized measurement technique.

### Relation of normal values to age

We used age-specific subgrouping in selected variables. This is consistent with current studies reporting normal values [2, 9]. Previously, age was intentionally not included in some analyses due to the uncertainty whether these effects represent true physiological aging [5]. However, a significant age effect was observed for several measurements in our and previous studies of normal populations [9–12]. Not correcting for age would lead to a large proportion of older patients without any apparent cardiovascular disease to fall outside the normal reference range.

### Reference limits for degrees of abnormality

Although several recent studies have defined the upper reference limits for many echocardiographic parameters [2, 9, 10], studies defining degrees of abnormality above this range are scarce [5]. In the absence of long-term prognostic data, a sample of the general population including patients with disease and using predefined percentiles is one of the possible approaches [4]. In our study, we had a general population sample, which allowed us to propose cut-offs using this methodology. These cut-offs are most relevant in variables where disease is prevalent in the general population, as is LV mass due to arterial hypertension. In these instances, degrees of abnormality can provide a sense of how extreme abnormal values are in a similar sense to degrees of abnormality provided in EACVI/ASE guidelines, which in some cases rely on multiples of standard deviation [1]. In other cases, low prevalence of disease in the general population makes difference between healthy and general populations negligible and these cut-offs only hypothesis-generating.

For some variables, most notably for LV ejection fraction and LA volume, cut-offs based on prognosis are well established and probably more useful [13, 14].

### LV dimensions, mass and wall thickness

We found similar cut-offs for both indexed and non-indexed LV end-diastolic dimensions compared to current recommendations [15]. Interestingly, our study shows a clinically relevant overestimation of dimension and wall thickness using M-mode imaging compared to the 2D method. This is an intriguing finding conflicting with some previous reports [16]. It is most strikingly shown in our LV mass measurement, where our M-mode derived cut-offs were higher than the currently recommended cut-off values (95 and 115 g/m^2^ for men and women, respectively) by almost 10 g/m^2^, while the 2D-derived limits were lower to a similar extent [15]. This can be visually appreciated in Additional file 1: Figures S1 and S2 and underscores the critical need to consider the measurement technique when evaluating LV mass. To the best of our knowledge, our study is the first to identify separate cut-offs for M-mode and 2D-derived LV mass derived from data obtained in such a large population sample. Furthermore, cut-offs for LV mass values significantly increased with age in our population, along with wall thickness. This trend has been shown in previous studies for both mass [9, 10] and wall thickness [10, 11]. Whether this phenomenon represents a genuine effect of aging or confounding by other factors associated with increasing age is impossible to differentiate based on our data [17].

### LV volumes and systolic function

Our LV volume cut-offs in apical views are similar to the current recommendations [1]. Ejection fraction cut-off was slightly lower than previously reported, but the known trend towards higher EF in females was numerically present in our population. Longitudinal systolic function assessed by s’ is in general consistent with values presented previously [18].

### LA volume, RV diameter and function

Left atrial volume reference limits were higher than the commonly accepted limit of 34 ml/m^2^ [4]. Epidemiological method of sampling our population could lead to these larger reference limits. Interestingly, our results are quite comparable to the more recently published NORRE data, that have shown an upper reference limit of 40.3 ml/m^2^ for both genders using the same methodology [9].

Right ventricular basal dimension was strongly dependent on gender, a significant difference not highlighted in the current recommendations, but shown in recent population studies [9, 10]. Interestingly, indexation to BSA was able to abolish this difference and a cut-off value of 23 mm/m^2^ seems reasonable as a sex-independent upper reference limit. A lower reference limit for TAPSE in our population was slightly higher than previously reported. Indexed right atrial diameters seemed comparable to previous recommendations [4].

## Limitations

A significant limitation of our study is the absence of deformation indices. Despite that, routine chamber evaluation is still often done using relatively simple techniques described in our paper.

Another limitation is that defining degrees of abnormality based on percentiles of the general population is mostly relevant when prevalence of pathology is sufficient in the general population, as is the case for LV mass. When there is a small number of patients with disease, we observed small to no differences in cut-offs differentiating mild, moderate and severe abnormality, especially in young patients where overall cardiovascular disease prevalence is low. In these cases, these limits should be used carefully, if at all. To address this issue, wherever the degrees of abnormality are too small and converged, we have reported that in the Result tables. Furthermore, absence of prognostic data is a limitation based on design of the Czech post-MONICA survey; however, a cross-sectional design is an established methodology for deriving normative values [15].

Simple indexing by BSA might be considered a limitation. However, it is the most commonly used indexation in echocardiography. Further analysis of anthropometric relations was beyond the scope of this manuscript.

## Conclusions

This analysis reports echocardiographic reference values for chamber dimensions and ventricular systolic function gained from the population-based Czech post-MONICA study, providing unique normative ranges applicable to Central European populations. The age and gender dependence of echocardiographic variables is described, as well as differences between M-mode and 2D imaging. Furthermore, cut-offs for mild, moderate and severe abnormalities based on percentiles of the general population are provided where feasible.

## Supplementary information

**Additional file 1: Figure S1.** Shows distribution of LV mass (both using M-mode and 2D imaging) and indexed LA volume distribution in both healthy and general cohort. It can be seen that distributions of most variables are significantly skewed. Furthermore, reference limits based on 95th percentile of normal population and 97.5th and 99th percentile of general populations are shown with points and annotated with values. Horizontal axis has been truncated at 150% of the 99th percentile of general population. Gaussian kernel density estimates are used for plotting. BSA, body surface area; LV, left ventricle. **Figure S2.** Showing distribution of indexed LV mass using different measurement methods by age and gender. Reference limits based on gender and age derived from quantile regression are shown. Severe abnormality is above 99th percentile of general population, moderate 97.5th – 99th percentile of general population and mild between 95th percentile of the healthy population and 97.5th percentile of general population. One female outlier from general population with LV mass over 250 g was excluded from the plotting but is included in the analyses. BSA, body surface area; LV, left ventricle. **Table S1.** Female reference limits by age - left ventricle. **Table S2.** Female reference limits by age – atria and aortic root. **Table S3.** Male reference limits by age – left ventricle. **Table S4.** Male reference limits by age – atria and aortic root. **Table S5.** Echocardiographic parameters for healthy population – left ventricle. **Table S6.** Echocardiographic parameters for healthy population – left atrium, right chambers and aorta.

**Additional file 2: Table S7.** Echocardiographic parameters for healthy population by gender and age – left ventricle. **Table S8.** Echocardiographic parameters for healthy population by gender and age – left atrium, right chambers and aorta. **Table S9.** Anthropometric variables for healthy population by age and gender.

## Data Availability

The data that support the findings of this study are available on reasonable request from the corresponding author, Linhart A. The data are not publicly available due to the containing information that could compromise the privacy of research participants.

## Abbreviations

BMI: Body mass index
BSA: Body surface area
LA: Left atrium
LV: Left ventricle
RV: Right ventricle
TAPSE: Tricuspid annular plane systolic excursion
