# Supplementary material for: Normative reference ranges for echocardiographic chamber dimensions in a healthy Central European population: results from the Czech post-MONICA survey
Source: Cardiovasc Ultrasound. 2019 Oct 30;17:22. doi: 10.1186/s12947-019-0172-0 (PMC6822480; doi:10.1186/s12947-019-0172-0)
Supplement: Supplementary file 1 — Additional file 1: Figure S1. Shows distribution of LV mass (both using M-mode and 2D imaging) and indexed LA volume distribution in both healthy and general cohort. It can be seen that distributions of most variables are significantly skewed. Furthermore, reference limits based on 95th percentile of normal population and 97.5th and 99th percentile of general populations are shown with points and annotated with values. Horizontal axis has been truncated at 150% of the 99th percentile of general population. Gaussian kernel density estimates are used for plotting. BSA, body surface area; LV, left ventricle. Figure S2. Showing distribution of indexed LV mass using different measurement methods by age and gender. Reference limits based on gender and age derived from quantile regression are shown. Severe abnormality is above 99th percentile of general population, moderate 97.5th – 99th percentile of general population and mild between 95th percentile of the healthy population and 97.5th percentile of general population. One female outlier from general population with LV mass over 250 g was excluded from the plotting but is included in the analyses. BSA, body surface area; LV, left ventricle. Table S1. Female reference limits by age - left ventricle. Table S2. Female reference limits by age – atria and aortic root. Table S3. Male reference limits by age – left ventricle. Table S4. Male reference limits by age – atria and aortic root. Table S5. Echocardiographic parameters for healthy population – left ventricle. Table S6. Echocardiographic parameters for healthy population – left atrium, right chambers and aorta. [file 12947_2019_172_MOESM1_ESM.docx]

**Figure S1.**

**
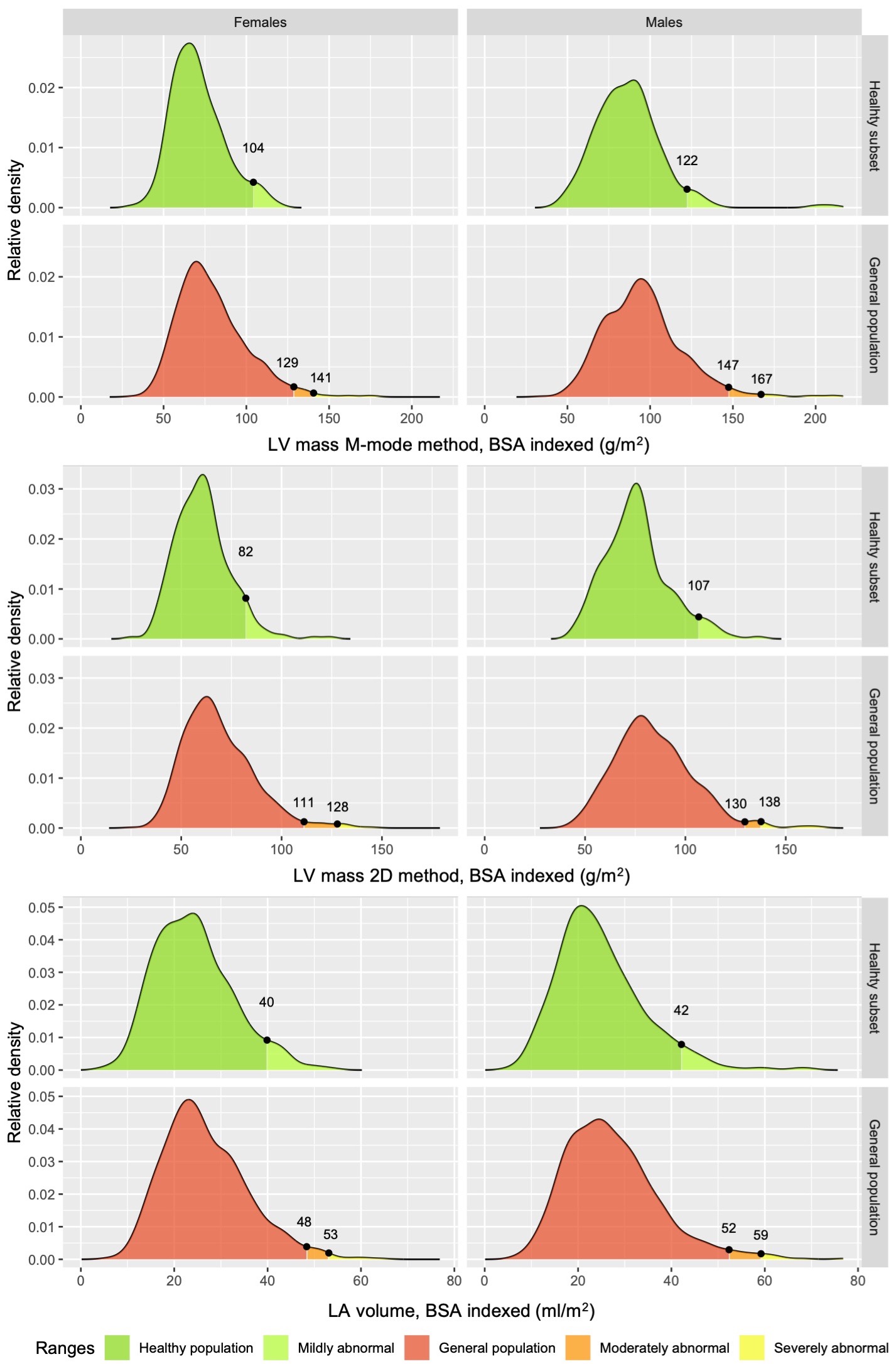
**

Figure S1 shows distribution of LV mass (both using M-mode and 2D imaging) and indexed LA volume distribution in both healthy and general cohort. It can be seen that distributions of most variables are significantly skewed. Furthermore, reference limits based on 95^th^ percentile of normal population and 97.5^th^ and 99^th^ percentile of general populations are shown with points and annotated with values. Horizontal axis has been truncated at 150% of the 99^th^ percentile of general population. Gaussian kernel density estimates are used for plotting. BSA, body surface area; LV, left ventricle.

**Figure S2.**

Figure S2 showing distribution of indexed LV mass using different measurement methods by age and gender. Reference limits based on gender and age derived from quantile regression are shown. Severe abnormality is above 99^th^ percentile of general population, moderate 97.5^th^ – 99^th^ percentile of general population and mild between 95^th^ percentile of the healthy population and 97.5^th^ percentile of general population. One female outlier from general population with LV mass over 250g was excluded from the plotting but is included in the analyses. BSA, body surface area; LV, left ventricle

**Table S1. Female reference limits by age - left ventricle**

| **Variable** | **Abnormal** | **Age by decade (years)** | | | |
| --- | --- | --- | --- | --- | --- |
|  |  | **35**  **(30 – 40)** | **45**  **(40 – 50)** | **55**  **(50 – 60)** | **65**  **(60 – 70)** |
| **LV dimensions** |  |  |  |  |  |
| **2D method** |  |  |  |  |  |
| LV end-diastolic diameter 2D (mm) | **Mildly** | **> 51.4** | **> 50.7** | **> 50.0** | **> 49.4** |
|  | Moderately | > 54.3 | > 55.3 | > 56.4 | > 57.4 |
|  | Severely | > 54.8 | > 56.1 | > 57.4 | > 58.7 |
| LV end-diastolic diameter 2D, BSA (mm) | **Mildly** | **> 30.1** | **> 29.7** | **> 29.2** | **> 28.8** |
|  | Moderately | (> 30.1)* | > 30.3 | > 30.6 | > 30.9 |
|  | Severely | (> 31.5)* | > 31.8 | > 32.2 | > 32.5 |
| **LV mass and wall thickness** |  |  |  |  |  |
| **2D method** |  |  |  |  |  |
| Interventricular septum 2D (mm) | **Mildly** | **> 9.5** | **> 10.0** | **> 10.5** | **> 11.0** |
|  | Moderately | > 10.8 | > 11.4 | > 12.1 | > 12.8 |
|  | Severely | > 11.6 | > 12.2 | > 12.9 | > 13.6 |
| Posterior wall 2D (mm) | **Mildly** | **> 8.7** | **> 9.2** | **> 9.6** | **> 10.0** |
|  | Moderately | > 9.7 | > 10.3 | > 10.8 | > 11.3 |
|  | Severely | > 10.5 | > 11.2 | > 11.9 | > 12.5 |
| LV mass 2D, BSA (g/m^2^) | **Mildly** | **> 78.7** | **> 82.9** | **> 87.1** | **> 91.4** |
|  | Moderately | > 87.9 | > 99.2 | > 110.5 | > 121.8 |
|  | Severely | > 94.0 | > 106.6 | > 119.2 | > 131.8 |
| **M-mode method** |  |  |  |  |  |
| Interventricular septum M‑mode (mm) | **Mildly** | **> 10.3** | **> 10.9** | **> 11.5** | **> 12.1** |
|  | Moderately | > 11.6 | > 12.2 | > 12.7 | > 13.3 |
|  | Severely | > 12.4 | > 13.1 | > 13.7 | > 14.4 |
| Posterior wall M‑mode (mm) | **Mildly** | **> 9.2** | **> 9.8** | **> 10.4** | **> 11.0** |
|  | Moderately | > 9.9 | > 10.7 | > 11.5 | > 12.3 |
|  | Severely | > 11.1 | > 11.8 | > 12.5 | > 13.2 |
| LV mass M‑mode, BSA (g/m^2^) | **Mildly** | **> 92.8** | **> 100.0** | **> 107.1** | **> 114.2** |
|  | Moderately | > 100.3 | > 113.4 | > 126.4 | > 139.5 |
|  | Severely | > 108.5 | > 126.5 | > 144.5 | > 162.5 |
| LV mass M-mode, heigth^2.7^ (g/m^2.7^) | **Mildly** | **> 41.7** | **> 45.9** | **> 50.2** | **> 54.4** |
|  | Moderately | > 50.6 | > 58.1 | > 65.6 | > 73.1 |
|  | Severely | > 54.2 | > 64.0 | > 73.9 | > 83.8 |
| **LV volume and function** |  |  |  |  |  |
| LV end-diastolic volume (ml) | **Mildly** | **> 118.8** | **> 112.3** | **> 105.7** | **> 99.1** |
|  | Moderately | > 130 | > 132.5 | > 135 | > 137.5 |
|  | Severely | > 143.6 | > 151.8 | > 159.9 | > 168.0 |
| LV end-systolic volume (ml) | **Mildly** | **> 46.8** | **> 43.6** | **> 40.3** | **> 37.1** |
|  | Moderately | > 52 | > 53.7 | > 55.5 | > 57.2 |
|  | Severely | > 64.3 | > 66.9 | > 69.5 | > 72.1 |
| LV end-systolic volume, BSA (ml/m^2^) | **Mildly** | **> 27.5** | **> 25.4** | **> 23.2** | **> 21.1** |
|  | Moderately | > 30.1 | > 30.3 | > 30.5 | > 30.7 |
|  | Severely | > 35.2 | > 36.4 | > 37.7 | > 39 |
| Mitral septal s' (cm/s) | **Mildly** | **< 6.0** | **< 5.8** | **< 5.5** | **< 5.3** |
|  | Moderately | < 5.9 | < 5.4 | < 5.0 | < 4.6 |
|  | Severely | < 5.8 | < 5.2 | < 4.7 | < 4.2 |
| Mitral lateral s' (cm/s) | **Mildly** | **< 6.4** | **< 6.1** | **< 5.8** | **< 5.5** |
|  | Moderately | < 6.1 | < 5.7 | < 5.3 | < 4.9 |
|  | Severely | < 5.9 | < 5.3 | < 4.8 | < 4.2 |

Reference values are based on the quantile regression model with age predictor value set at median for each age group, i.e. age 35 for age group 30 – 40. * - Reference value set as same as reference value of abnormality, see Limitations for discussion of collapsing degrees of abnormality. BSA, body surface area; LV, left ventricle.

**Table S2. Female reference limits by age – atria and aortic root**

| **Variable** | **Abnormal** | **Age by decade (years)** | | | |
| --- | --- | --- | --- | --- | --- |
|  |  | **35**  **(30 – 40)** | **45**  **(40 – 50)** | **55**  **(50 – 60)** | **65**  **(60 – 70)** |
| **Left atrium** |  |  |  |  |  |
| LA diameter M-mode, BSA (mm/m^2^) | **Mildly** | **> 23.2** | **> 24.2** | **> 25.2** | **> 26.2** |
|  | Moderately | > 23.9 | > 25.0 | > 26.1 | > 27.2 |
|  | Severely | > 24.5 | > 25.8 | > 27.2 | > 28.5 |
| **Right atrium** |  |  |  |  |  |
| RA vertical diameter, BSA (mm/m^2^) | **Mildly** | **> 29.4** | **> 29.9** | **> 30.5** | **> 31.1** |
|  | Moderately | > 29.5 | > 30.4 | > 31.3 | > 32.3 |
|  | Severely | > 29.8 | > 30.9 | > 32.0 | > 33.1 |
| **Aorta** |  |  |  |  |  |
| Aortic root, BSA (mm) | Mildly | > 19.1 | > 19.5 | > 20.0 | > 20.4 |
|  | Moderately | > 19.9 | > 20.3 | > 20.8 | > 21.2 |
|  | Severely | > 20.5 | > 21.2 | > 21.8 | > 22.4 |

Reference values are based on the quantile regression model with age predictor value set at median for each age group, i.e. age 35 for age group 30 – 40. * - Reference value set as same as reference value of abnormality, see Limitations for discussion of collapsing degrees of abnormality. BSA, body surface area; LA, left atrium; RA, right atrium; TAPSE, tricuspid annular systolic plane excursion.

**Table S3. Male reference limits by age – left ventricle**

| **Variable** | **Abnormal** | **Age by decade (years)** | | | |
| --- | --- | --- | --- | --- | --- |
|  |  | **35**  **(30 – 40)** | **45**  **(40 – 50)** | **55**  **(50 – 60)** | **65**  **(60 – 70)** |
| **LV dimensions** |  |  |  |  |  |
| **2D method** |  |  |  |  |  |
| LV end-diastolic diameter 2D (mm) | **Mildly** | **> 57.0** | **> 56.3** | **> 55.7** | **> 55.0** |
|  | Moderately | > 58.6 | > 59.7 | > 60.7 | > 61.8 |
|  | Severely | > 62.3 | > 63.6 | > 64.9 | > 66.2 |
| LV end-diastolic diameter 2D, BSA (mm) | **Mildly** | **> 28.6** | **> 28.2** | **> 27.8** | **> 27.4** |
|  | Moderately | > 29.1 | > 29.4 | > 29.7 | > 30.0 |
|  | Severely | > 30.4 | > 30.8 | > 31.2 | > 31.5 |
| **LV mass and wall thickness** |  |  |  |  |  |
| **2D method** |  |  |  |  |  |
| Interventricular septum 2D (mm) | **Mildly** | **> 10.9** | **> 11.4** | **> 11.8** | **> 12.3** |
|  | Moderately | > 12.3 | > 13.0 | > 13.7 | > 14.3 |
|  | Severely | > 13.7 | > 14.4 | > 15.1 | > 15.8 |
| Posterior wall 2D (mm) | **Mildly** | **> 10.2** | **> 10.7** | **> 11.1** | **> 11.5** |
|  | Moderately | > 11.2 | > 11.7 | > 12.2 | > 12.8 |
|  | Severely | > 11.7 | > 12.3 | > 13.0 | > 13.6 |
| LV mass 2D, BSA (g/m^2^) | **Mildly** | **> 102.2** | **> 106.4** | **> 110.6** | **> 114.9** |
|  | Moderately | > 107.8 | > 119.1 | > 130.3 | > 141.6 |
|  | Severely | > 124.3 | > 136.9 | > 149.5 | > 162.1 |
| **M-mode method** |  |  |  |  |  |
| Interventricular septum M‑mode (mm) | **Mildly** | **> 12.0** | **> 12.6** | **> 13.2** | **> 13.8** |
|  | Moderately | > 13.0 | > 13.6 | > 14.1 | > 14.7 |
|  | Severely | > 14.3 | > 14.9 | > 15.6 | > 16.2 |
| Posterior wall M‑mode (mm) | **Mildly** | **> 10.7** | **> 11.3** | **> 11.9** | **> 12.5** |
|  | Moderately | > 11.4 | > 12.2 | > 13.0 | > 13.8 |
|  | Severely | > 12.6 | > 13.2 | > 13.9 | > 14.6 |
| LV mass M‑mode, BSA (g/m^2^) | **Mildly** | **> 115.4** | **> 122.5** | **> 129.6** | **> 136.8** |
|  | Moderately | > 122.5 | > 135.6 | > 148.6 | > 161.7 |
|  | Severely | > 137.2 | > 155.3 | > 173.3 | > 191.3 |
| LV mass M-mode, heigth^2.7^ (g/m^2.7^) | **Mildly** | **> 46.5** | **> 50.8** | **> 55** | **> 59.2** |
|  | Moderately | > 54.2 | > 61.7 | > 69.2 | > 76.6 |
|  | Severely | > 58.6 | > 68.4 | > 78.3 | > 88.1 |
| **LV volumes and function** |  |  |  |  |  |
| LV end-diastolic volume (ml) | **Mildly** | **> 154.2** | **> 147.6** | **> 141.0** | **> 134.4** |
|  | Moderately | > 175.6 | > 178.1 | > 180.6 | > 183.1 |
|  | Severely | > 192.0 | > 200.1 | > 208.3 | > 216.4 |
| LV end-systolic volume (ml) | **Mildly** | **> 70.1** | **> 66.6** | **> 63.0** | **> 59.4** |
|  | Moderately | > 78.7 | > 80.2 | > 81.7 | > 83.2 |
|  | Severely | > 90.1 | > 92.7 | > 95.3 | > 97.9 |
| LV end-systolic volume, BSA (ml/m^2^) | **Mildly** | **> 33.9** | **> 31.7** | **> 29.6** | **> 27.5** |
|  | Moderately | > 38.4 | > 38.6 | > 38.8 | > 39 |
|  | Severely | > 43.5 | > 44.8 | > 46 | > 47.3 |
| Mitral septal s' (cm/s) | **Mildly** | **< 5.9** | **< 5.7** | **< 5.4** | **< 5.2** |
|  | Moderately | < 5.8 | < 5.4 | < 4.9 | < 4.5 |
|  | Severely | < 5.3 | < 4.7 | < 4.2 | < 3.7 |
| Mitral lateral s' (cm/s) | **Mildly** | **< 6.7** | **< 6.4** | **< 6.1** | **< 5.8** |
|  | Moderately | < 5.9 | < 5.5 | < 5.1 | < 4.7 |
|  | Severely | < 5.8 | < 5.2 | < 4.6 | < 4.1 |

Reference values are based on the quantile regression model with age predictor value set at median for each age group, i.e. age 35 for age group 30 – 40. BSA, body surface area; LV, left ventricle.

**Table S4. Male reference limits by age – atria and aortic root**

| **Variable** | **Abnormal** | **Age by decade (years)** | | | |
| --- | --- | --- | --- | --- | --- |
|  |  | **35**  **(30 – 40)** | **45**  **(40 – 50)** | **55**  **(50 – 60)** | **65**  **(60 – 70)** |
| **Left atrium** |  |  |  |  |  |
| LA diameter M-mode, BSA (mm/m^2^) | **Mildly** | **> 22.0** | **> 23.0** | **> 24.0** | **> 25.0** |
|  | Moderately | > 23.3 | > 24.3 | > 25.4 | > 26.5 |
|  | Severely | > 23.7 | > 25.1 | > 26.4 | > 27.7 |
| **Right atrium** |  |  |  |  |  |
| RA vertical diameter, BSA (mm/m^2^) | **Mildly** | **> 27.8** | **> 28.3** | **> 28.9** | **> 29.5** |
|  | Moderately | > 28.1 | > 29.0 | > 29.9 | > 30.8 |
|  | Severely | > 29.2 | > 30.3 | > 31.4 | > 32.4 |
| **Aorta** |  |  |  |  |  |
| Aortic root, BSA (mm) | Mildly | > 18.9 | > 19.3 | > 19.7 | > 20.1 |
|  | Moderately | > 19.4 | > 19.9 | > 20.3 | > 20.8 |
|  | Severely | > 19.8 | > 20.4 | > 21.1 | > 21.7 |

Reference values are based on the quantile regression model with age predictor value set at median for each age group, i.e. age 35 for age group 30 – 40. BSA, body surface area; LA, left atrium; RA, right atrium; TAPSE – tricuspid annular systolic plane excursion.

**Table S5. Echocardiographic parameters for healthy population – left ventricle.**

|  | **Males (n = 247)** | | |  | **Females (n = 328)** | | |
| --- | --- | --- | --- | --- | --- | --- | --- |
| **Variable** | **Mean**  **± SD** | **95%**  **Range** | **Median [25^th^,75^th^]** |  | **Mean**  **± SD** | **95%**  **Range** | **Median [25^th^,75^th^]** |
| **LV dimensions** |  |  |  |  |  |  |  |
| **2D method** |  |  |  |  |  |  |  |
| LV end-diastolic diameter (mm) | 49.4 ± 4.4 | 40.7 - 58.0 | 49.3 [46.7,52.3] |  | 44.5 ± 4.0 | 36.7 - 52.3 | 44.4 [42.0,47.0] |
| LV end-diastolic diameter, BSA (mm/m^2^) | 24.6 ± 2.3 | 20.1 - 29.1 | 24.5 [23.3,26.0] |  | 25.9 ± 2.4 | 21.3 - 30.5 | 26.0 [24.5,27.5] |
| **M-mode method** |  |  |  |  |  |  |  |
| LV end-diastolic diameter (mm) | 52.6 ± 4.5 | 43.8 - 61.5 | 52.3 [49.3,56.0] |  | 47.5 ± 4.2 | 39.2 - 55.7 | 47.3 [44.7,50.6] |
| LV end-diastolic diameter, BSA (mm/m^2^) | 26.2 ± 2.4 | 21.4 - 31.0 | 25.9 [24.4,27.6] |  | 27.7 ± 2.5 | 22.8 - 32.6 | 27.7 [26.0,29.3] |
| LV end-systolic diameter (mm) | 32.6 ± 4.6 | 23.7 - 41.6 | 32.7 [30.0,35.7] |  | 28.6 ± 4.2 | 20.5 - 36.8 | 28.3 [26.0,31.3] |
| LV end-systolic diameter, BSA (mm/m^2^) | 16.3 ± 2.4 | 11.6 - 20.9 | 16.1 [14.6,17.6] |  | 16.7 ± 2.3 | 12.1 - 21.3 | 16.5 [15.2,18.1] |
| **LV mass and wall thickness** |  |  |  |  |  |  |  |
| **2D method** |  |  |  |  |  |  |  |
| Interventricular septum (mm) | 9.3 ± 1.4 | 6.6 - 12.0 | 9.0 [8.3,10.0] |  | 9.3 ± 1.4 | 6.6 - 12.0 | 9.0 [8.3,10.0] |
| Posterior wall (mm) | 8.5 ± 1.3 | 6.0 - 11.0 | 8.3 [7.7,9.3] |  | 7.3 ± 1.2 | 5.0 - 9.6 | 7.3 [6.3,8.0] |
| LV mass, BSA (g/m^2^) | 75.8 ± 15.8 | 44.7 - 106.8 | 75.1 [65.3,82.8] |  | 60.5 ± 13.3 | 34.4 - 86.6 | 59.5 [51.1,67.1] |
| **M-mode method** |  |  |  |  |  |  |  |
| Interventricular septum (mm) | 9.5 ± 1.7 | 6.1 - 12.9 | 9.3 [8.3,10.7] |  | 8.3 ± 1.5 | 5.3 - 11.3 | 8.3 [7.0,9.3] |
| Posterior wall (mm) | 8.6 ± 1.4 | 5.8 - 11.4 | 8.5 [7.7,9.3] |  | 7.4 ± 1.3 | 4.8 - 10.1 | 7.3 [6.3,8.3] |
| LV mass, BSA (g/m^2^) | 86.9 ± 20.9 | 45.9 - 128.0 | 86.3 [73.3,96.6] |  | 70.8 ± 15.7 | 39.9 - 101.6 | 68.5 [59.4,80.1] |
| LV mass, height^2.7^ (g/m) | 36.1 ± 9.7 | 17.2 - 55.1 | 35.6 [30.1,40.8] |  | 31.0 ± 7.9 | 15.6 - 46.5 | 29.6 [25.5,35.1] |
| **LV volumes and function** |  |  |  |  |  |  |  |
| LV end-diastolic volume (ml) | 105.5 ± 25.1 | 56.3 - 154.8 | 105.3 [90.1,120.7] |  | 79.4 ± 18.6 | 43.1 - 115.8 | 78.2 [66.7,89.8] |
| LV end-diastolic volume, BSA (ml/m^2^) | 52.3 ± 11.7 | 29.4 - 75.3 | 51.2 [44.5,59.5] |  | 46.2 ± 10.2 | 26.2 - 66.1 | 45.9 [39.6,52.6] |
| LV end-systolic volume (ml) | 40.5 ± 14.0 | 13.1 - 67.9 | 38.3 [31.3,48.0] |  | 29.0 ± 8.6 | 12.2 - 45.8 | 28.0 [23.3,34.0] |
| LV end-systolic volume, BSA (ml/m^2^) | 20.1 ± 6.8 | 6.8 - 33.4 | 19.3 [15.8,22.9] |  | 16.9 ± 4.8 | 7.4 - 26.4 | 16.3 [13.4,19.6] |
| LV ejection fraction (%) | 61.9 ± 7.3 | 47.6 - 76.3 | 61.8 [57.3,66.5] |  | 63.4 ± 6.4 | 50.9 - 75.8 | 63.3 [59.4,67.3] |
| Mitral septal s' (cm/s) | 7.9 ± 1.4 | 5.1 - 10.6 | 8.0 [7.0,8.7] |  | 7.9 ± 1.4 | 5.2 - 10.5 | 8.0 [7.0,9.0] |
| Mitral lateral s' (cm/s) | 9.7 ± 2.4 | 5.0 - 14.5 | 9.3 [8.0,11.0] |  | 9.6 ± 2.4 | 5.0 - 14.2 | 9.3 [8.0,11.0] |

The 95% range is derived based on mean ± 1.96 times standard deviation. However, due to non-normal distributions, these ranges should be used cautiously. 25^th^ to 75^th^ percentile is shown along with the median value. BSA, body surface area; LV, left ventricle; SD, standard deviation.

**Table S6. Echocardiographic parameters for healthy population – left atrium, right chambers and aorta.**

|  | **Males (n = 247)** | | |  | **Females (n = 328)** | | |
| --- | --- | --- | --- | --- | --- | --- | --- |
| **Variable** | **Mean**  **± SD** | **95%**  **Range** | **Median [25^th^,75^th^]** |  | **Mean**  **± SD** | **95%**  **Range** | **Median [25^th^,75^th^]** |
| **Left atrium** |  |  |  |  |  |  |  |
| LA diameter M-mode (mm) | 38.2 ± 4.6 | 29.3 - 47.2 | 38.3 [35.3,41.3] |  | 34.3 ± 4.0 | 26.6 - 42.1 | 34.3 [31.7,37.0] |
| LA diameter M-mode, BSA (mm/m^2^) | 19.1 ± 2.3 | 14.6 - 23.5 | 19.1 [17.6,20.2] |  | 20.0 ± 2.4 | 15.3 - 24.7 | 19.8 [18.4,21.5] |
| LA vertical diameter (mm) | 49.3 ± 6.3 | 36.9 - 61.6 | 49.0 [45.3,52.3] |  | 46.1 ± 6.0 | 34.3 - 57.9 | 46.0 [42.0,50.3] |
| LA horizontal diameter (mm) | 39.4 ± 5.0 | 29.6 - 49.1 | 39.0 [35.7,43.0] |  | 37.4 ± 4.5 | 28.6 - 46.3 | 37.0 [34.3,40.3] |
| LA volume (ml) | 50.1 ± 19.6 | 11.7 - 88.5 | 47.0 [36.0,60.3] |  | 41.9 ± 14.6 | 13.2 - 70.6 | 40.0 [31.0,50.6] |
| LA volume, BSA (ml/m^2^) | 24.8 ± 9.2 | 6.8 - 42.8 | 23.4 [18.5,29.6] |  | 24.3 ± 8.2 | 8.1 - 40.4 | 23.4 [18.2,29.4] |
| **Right ventricle** |  |  |  |  |  |  |  |
| RV basal diameter (mm) | 36.7 ± 5.3 | 26.3 - 47.1 | 36.4 [33.3,40.0] |  | 31.3 ± 4.5 | 22.6 - 40.1 | 31.0 [28.7,34.0] |
| RV basal diameter, BSA (mm/m^2^) | 18.3 ± 2.7 | 12.9 - 23.6 | 18.0 [16.6,20.1] |  | 18.2 ± 2.7 | 13.0 - 23.5 | 18.3 [16.6,20.0] |
| Tricuspid s’ (m/s) | 12.5 ± 2.5 | 7.7 - 17.3 | 12.3 [11.0,14.0] |  | 12.7 ± 2.3 | 8.2 - 17.3 | 12.3 [11.0,14.0] |
| TAPSE (mm) | 24.3 ± 3.5 | 17.5 - 31.1 | 24.0 [21.8,26.3] |  | 24.5 ± 3.3 | 18.1 - 30.9 | 24.3 [22.0,26.8] |
| **Right atrium** |  |  |  |  |  |  |  |
| RA vertical diameter (mm) | 48.1 ± 5.2 | 37.8 - 58.3 | 48.0 [45.0,51.3] |  | 43.4 ± 4.7 | 34.2 - 52.7 | 43.7 [40.7,46.0] |
| RA horizontal diameter (mm) | 39.5 ± 6.0 | 27.9 - 51.2 | 39.0 [35.3,43.0] |  | 34.2 ± 4.5 | 25.4 - 43.1 | 33.7 [31.3,37.0] |
| RA vertical diameter, BSA (mm/m^2^) | 23.9 ± 2.6 | 18.7 - 29.1 | 23.8 [22.3,25.5] |  | 25.3 ± 2.7 | 20.0 - 30.5 | 25.3 [23.4,27.1] |
| RA horizontal diameter, BSA (mm/m^2^) | 19.7 ± 3.0 | 13.8 - 25.6 | 19.6 [17.6,21.8] |  | 20.0 ± 2.8 | 14.5 - 25.4 | 19.7 [18.1,21.7] |
| **Aorta** |  |  |  |  |  |  |  |
| Aortic root (mm) | 32.7 ± 3.7 | 25.5 - 39.9 | 32.3 [29.7,35.0] |  | 28.5 ± 3.2 | 22.3 - 34.8 | 28.7 [26.3,30.7] |
| Aortic root, BSA (mm/m2) | 16.3 ± 1.9 | 12.7 - 19.9 | 16.3 [14.9,17.5] |  | 16.6 ± 1.9 | 12.9 - 20.3 | 16.5 [15.3,17.7] |

The 95% range is derived based on mean ± 1.96 times standard deviation. However, due to non-normal distributions, these ranges should be used cautiously. 25^th^ to 75^th^ percentile is shown along with the median value. BSA, body surface area; LA, left atrium; RA, right atrium; RV, right ventricle; TAPSE, tricuspid annular systolic plane excursion; SD, standard deviation.
