# Supplementary material for: Normative reference ranges for echocardiographic chamber dimensions in a healthy Central European population: results from the Czech post-MONICA survey
Source: Cardiovasc Ultrasound. 2019 Oct 30;17:22. doi: 10.1186/s12947-019-0172-0 (PMC6822480; doi:10.1186/s12947-019-0172-0)
Supplement: Supplementary file 2 — Additional file 2: Table S7. Echocardiographic parameters for healthy population by gender and age – left ventricle. Table S8. Echocardiographic parameters for healthy population by gender and age – left atrium, right chambers and aorta. Table S9. Anthropometric variables for healthy population by age and gender. [file 12947_2019_172_MOESM2_ESM.docx]

**Table S7. Echocardiographic parameters for healthy population by gender and age – left ventricle.**

| **Gender** | **Females (n = 328)** | | | | | | **Males (n =247)** | | | | | | **Models*** | |
| --- | --- | --- | --- | --- | --- | --- | --- | --- | --- | --- | --- | --- | --- | --- |
| **Age group** | **Under 40** | | **40-60** | | **Over 60** | | **Under 40** | | **40-60** | | **Over 60** | |  |  |
| **Variable** | **Mean ± SD** | **Median [25^th^,75^th^]** | **Mean ± SD** | **Median [25^th^,75^th^]** | **Mean ± SD** | **Median [25^th^,75^th^]** | **Mean ± SD** | **Median [25^th^,75^th^]** | **Mean ± SD** | **Median [25^th^,75^th^]** | **Mean ± SD** | **Median [25^th^,75^th^]** | **Age**  **p** | **Sex**  **p** |
| **LV dimensions** |  |  |  |  |  |  |  |  |  |  |  |  |  |  |
| **2D method** |  |  |  |  |  |  |  |  |  |  |  |  |  |  |
| LV end-diastolic diameter (mm) | 44.7 ± 3.9 | 44.3 [42.0,47.3] | 44.5 ± 3.9 | 44.7 [42.3,46.7] | 43.3 ± 5.1 | 44.7 [39.5,47.2] | 50.1 ± 4.1 | 50.0 [47.0,52.7] | 49.2 ± 4.6 | 49.3 [47.0,52.2] | 47.0 ± 3.9 | 47.0 [44.5,50.0] | 0.284 | < 0.001 |
| LV end-diastolic diameter, BSA (mm/m^2^) | 26.2 ± 2.2 | 26.3 [24.7,27.7] | 25.8 ± 2.4 | 25.9 [24.5,27.1] | 25.5 ± 2.9 | 25.9 [23.2,27.7] | 24.7 ± 2.2 | 24.5 [23.3,25.9] | 24.6 ± 2.5 | 24.5 [23.5,26.1] | 24.2 ± 2.0 | 24.8 [23.2,26.0] | 0.352 | < 0.001 |
| **M-mode method** |  |  |  |  |  |  |  |  |  |  |  |  |  |  |
| LV end-diastolic diameter (mm) | 47.6 ± 3.8 | 47.7 [44.9,50.1] | 47.4 ± 4.5 | 47.0 [44.7,51.0] | 47.4 ± 4.0 | 47.8 [44.0,49.8] | 52.6 ± 4.2 | 52.3 [49.3,55.5] | 52.5 ± 4.8 | 52.0 [49.0,56.0] | 53.7 ± 4.8 | 53.3 [50.2,56.4] | 0.006 | < 0.001 |
| LV end-diastolic diameter, BSA (mm/m^2^) | 27.9 ± 2.3 | 27.9 [26.4,29.3] | 27.5 ± 2.6 | 27.4 [25.9,29.4] | 28.0 ± 2.7 | 28.2 [26.7,29.2] | 26.0 ± 2.4 | 25.7 [24.4,27.4] | 26.2 ± 2.4 | 26.0 [24.4,27.6] | 27.7 ± 2.3 | 27.6 [26.7,28.9] | 0.068 | < 0.001 |
| LV end-systolic diameter (mm) | 29.3 ± 3.6 | 29.0 [27.0,31.3] | 28.2 ± 4.2 | 27.9 [25.7,31.3] | 27.9 ± 5.8 | 27.0 [24.6,28.6] | 32.9 ± 3.9 | 32.8 [30.7,36.0] | 32.3 ± 5.3 | 32.3 [29.3,35.3] | 32.8 ± 4.5 | 32.3 [30.3,35.0] | 0.203 | < 0.001 |
| LV end-systolic diameter, BSA (mm/m^2^) | 17.2 ± 2.1 | 17.2 [15.7,18.5] | 16.4 ± 2.4 | 16.3 [14.9,17.8] | 16.4 ± 2.7 | 16.0 [14.8,17.3] | 16.3 ± 2.1 | 16.0 [14.8,17.8] | 16.1 ± 2.6 | 16.1 [14.5,17.4] | 16.9 ± 2.2 | 17.1 [15.8,17.3] | 0.212 | 0.019 |
| **LV mass and wall thickness** |  |  |  |  |  |  |  |  |  |  |  |  |  |  |
| **2D method** |  |  |  |  |  |  |  |  |  |  |  |  |  |  |
| Interventricular septum (mm) | 7.5 ± 1.1 | 7.3 [6.7,8.0] | 8.1 ± 1.2 | 8.0 [7.3,8.7] | 8.8 ± 1.4 | 8.5 [8.0,10.1] | 8.9 ± 1.3 | 8.7 [8.0,9.7] | 9.5 ± 1.3 | 9.3 [8.7,10.3] | 10.3 ± 1.4 | 10.3 [9.2,10.8] | < 0.001 | < 0.001 |
| Posterior wall (mm) | 6.8 ± 1.0 | 6.8 [6.0,7.4] | 7.5 ± 1.2 | 7.7 [6.7,8.3] | 8.2 ± 1.0 | 8.5 [7.3,9.0] | 8.3 ± 1.1 | 8.3 [7.3,9.0] | 8.5 ± 1.3 | 8.3 [7.7,9.3] | 9.4 ± 1.4 | 9.0 [8.7,10.2] | < 0.001 | < 0.001 |
| LV mass, BSA (g/m^2^) | 56.7 ± 12.4 | 55.2 [47.3,63.2] | 62.1 ± 13.1 | 61.2 [53.6,68.7] | 67.9 ± 14.2 | 68.1 [58.1,73.9] | 73.3 ± 13.6 | 73.0 [64.8,79.8] | 76.7 ± 17.2 | 75.2 [65.6,84.9] | 83.1 ± 17.1 | 77.8 [72.0,94.5] | < 0.001 | < 0.001 |
| **M-mode method** |  |  |  |  |  |  |  |  |  |  |  |  |  |  |
| Interventricular septum (mm) | 7.9 ± 1.5 | 8.0 [6.9,9.0] | 8.5 ± 1.5 | 8.6 [7.3,9.7] | 8.8 ± 1.7 | 9.0 [7.6,10.1] | 9.1 ± 1.5 | 9.0 [8.0,10.3] | 9.7 ± 1.8 | 9.7 [8.7,10.7] | 10.7 ± 2.0 | 10.7 [9.8,11.3] | < 0.001 | < 0.001 |
| Posterior wall (mm) | 6.9 ± 1.1 | 7.0 [6.2,7.7] | 7.7 ± 1.3 | 7.7 [6.7,8.5] | 8.7 ± 1.3 | 8.4 [7.7,9.3] | 8.4 ± 1.2 | 8.3 [7.7,9.3] | 8.6 ± 1.5 | 8.6 [7.7,9.3] | 9.8 ± 1.9 | 9.4 [8.4,10.7] | < 0.001 | < 0.001 |
| LV mass, BSA (g/m^2^) | 66.1 ± 14.0 | 63.8 [55.8,74.6] | 72.6 ± 15.8 | 70.5 [61.5,82.0] | 81.8 ± 16.4 | 79.1 [68.4,91.7] | 82.0 ± 15.9 | 81.7 [68.9,92.5] | 87.8 ± 18.5 | 89.0 [75.4,99.9] | 111.1 ± 36.8 | 105.1 [91.8,119.3] | < 0.001 | < 0.001 |
| LV mass, height^2.7^ (g/m) | 28.1 ± 6.6 | 27.6 [23.5,31.7] | 32.2 ± 7.8 | 31.5 [26.5,36.2] | 38.1 ± 8.7 | 36.6 [31.3,42.9] | 33.6 ± 7.0 | 33.6 [28.6,37.2] | 36.5 ± 8.3 | 36.8 [30.8,42.0] | 48.3 ± 17.7 | 42.3 [38.7,52.2] | < 0.001 | < 0.001 |
| **LV volumes and function** |  |  |  |  |  |  |  |  |  |  |  |  |  |  |
| LV end-diastolic volume (ml) | 80.5 ± 20.0 | 78.7 [66.0,91.7] | 79.8 ± 16.9 | 79.0 [70.3,89.2] | 72.3 ± 21.1 | 72.0 [61.8,83.2] | 112.8 ± 26.1 | 112.3 [92.0,125.7] | 100.8 ± 22.9 | 100.0 [87.0,118.3] | 94.7 ± 21.6 | 94.7 [74.4,110.0] | < 0.001 | < 0.001 |
| LV end-diastolic volume, BSA (ml/m^2^) | 47.0 ± 10.6 | 46.2 [39.2,53.5] | 46.1 ± 9.4 | 46.0 [40.8,52.1] | 42.7 ± 12.5 | 41.6 [34.0,46.9] | 55.2 ± 12.3 | 53.8 [45.8,62.2] | 50.2 ± 10.8 | 49.9 [43.2,58.3] | 48.7 ± 10.6 | 49.4 [40.1,55.8] | < 0.001 | < 0.001 |
| LV end-systolic volume (ml) | 30.1 ± 9.2 | 30.3 [24.2,35.2] | 28.8 ± 7.9 | 27.3 [23.3,33.5] | 25.6 ± 8.6 | 23.3 [18.9,30.1] | 45.8 ± 14.6 | 42.3 [35.0,54.7] | 37.0 ± 12.1 | 36.0 [29.3,42.7] | 32.7 ± 10.1 | 33.2 [24.5,38.9] | < 0.001 | < 0.001 |
| LV end-systolic volume, BSA (ml/m^2^) | 17.6 ± 5.0 | 17.5 [14.1,19.8] | 16.7 ± 4.6 | 15.9 [13.3,19.6] | 15.0 ± 4.9 | 14.8 [11.6,17.0] | 22.4 ± 7.2 | 21.2 [17.5,25.6] | 18.5 ± 5.9 | 18.0 [14.8,21.6] | 16.8 ± 5.0 | 17.1 [13.1,20.4] | < 0.001 | < 0.001 |
| LV ejection fraction (%) | 62.6 ± 6.3 | 63.1 [58.5,66.4] | 63.8 ± 6.3 | 63.2 [59.8,68.3] | 64.4 ± 6.8 | 63.8 [59.8,69.5] | 59.7 ± 6.7 | 60.2 [57.0,63.8] | 63.3 ± 7.6 | 63.5 [57.3,69.1] | 65.7 ± 5.7 | 65.3 [62.2,68.9] | 0.012 | < 0.001 |
| Mitral septal s' (cm/s) | 8.2 ± 1.3 | 8.0 [7.3,9.0] | 7.7 ± 1.4 | 7.7 [6.7,8.6] | 7.4 ± 1.3 | 7.7 [6.3,8.2] | 7.8 ± 1.3 | 7.7 [7.0,8.6] | 8.0 ± 1.5 | 8.0 [7.0,9.0] | 7.4 ± 1.3 | 7.3 [6.7,8.0] | < 0.001 | 0.014 |
| Mitral lateral s' (cm/s) | 10.7 ± 2.3 | 11.0 [9.3,12.0] | 9.0 ± 2.1 | 9.0 [7.6,10.2] | 8.1 ± 1.8 | 8.0 [7.0,9.2] | 9.8 ± 2.5 | 9.7 [8.0,11.0] | 9.9 ± 2.4 | 9.3 [8.2,11.0] | 8.5 ± 1.7 | 8.0 [7.3,9.7] | < 0.001 | 0.133 |

Summary values are shown by age and gender subgroups. Due to limited sample size in subgroups and non-normal distributions, parametric range based on ± 1.96 times SD value should be used very cautiously and nonparametric values based on a quantile model from Tables S1-S4 should be preferred. * p-value is based on a quantile regression model estimating median value with age and gender as independent predictors. 25^th^ to 75^th^ percentile is shown along with the median value. BSA, body surface area; LV, left ventricle; SD, standard deviation.

**Table S8. Echocardiographic parameters for healthy population by gender and age – left atrium, right chambers and aorta.**

| **Gender** | **Females (n = 328)** | | | | | | **Males (n =247)** | | | | | | **Models*** | |
| --- | --- | --- | --- | --- | --- | --- | --- | --- | --- | --- | --- | --- | --- | --- |
| **Age group** | **Under 40** | | **40-60** | | **Over 60** | | **Under 40** | | **40-60** | | **Over 60** | |  |  |
| **Variable** | **Mean ± SD** | **Median [25^th^,75^th^]** | **Mean ± SD** | **Median [25^th^,75^th^]** | **Mean ± SD** | **Median [25^th^,75^th^]** | **Mean ± SD** | **Median [25^th^,75^th^]** | **Mean ± SD** | **Median [25^th^,75^th^]** | **Mean ± SD** | **Median [25^th^,75^th^]** | **Age**  **p** | **Sex**  **p** |
| **Left atrium** |  |  |  |  |  |  |  |  |  |  |  |  |  |  |
| LA diameter M-mode (mm) | 33.3 ± 3.5 | 33.3 [30.9,35.7] | 34.9 ± 4.2 | 34.7 [32.4,37.7] | 37.0 ± 3.2 | 36.0 [35.3,39.0] | 37.3 ± 4.5 | 37.7 [34.3,40.7] | 38.7 ± 4.4 | 39.2 [35.8,41.3] | 42.1 ± 4.7 | 42.3 [39.7,44.1] | < 0.001 | < 0.001 |
| LA diameter M-mode, BSA (mm/m^2^) | 19.5 ± 2.0 | 19.4 [18.0,20.9] | 20.2 ± 2.6 | 20.2 [18.4,21.8] | 21.4 ± 2.3 | 21.8 [19.9,22.8] | 18.4 ± 2.1 | 18.3 [17.0,19.7] | 19.5 ± 2.1 | 19.5 [18.0,20.5] | 21.7 ± 2.5 | 21.5 [19.9,23.3] | < 0.001 | < 0.001 |
| LA vertical diameter (mm) | 45.0 ± 6.4 | 44.7 [40.4,49.8] | 47.0 ± 5.6 | 47.0 [43.3,51.3] | 45.4 ± 6.2 | 46.3 [41.0,48.6] | 49.0 ± 7.2 | 48.0 [44.3,52.3] | 48.9 ± 5.2 | 49.0 [45.7,52.0] | 51.6 ± 6.4 | 51.0 [46.3,55.1] | < 0.001 | < 0.001 |
| LA horizontal diameter (mm) | 36.0 ± 3.9 | 35.8 [33.7,38.7] | 38.6 ± 4.5 | 38.0 [35.3,41.3] | 36.9 ± 5.4 | 36.7 [33.3,40.1] | 39.3 ± 5.1 | 39.0 [35.7,43.0] | 38.8 ± 4.7 | 38.7 [35.3,42.0] | 42.6 ± 4.6 | 42.8 [39.8,44.4] | < 0.001 | < 0.001 |
| LA volume (ml) | 37.0 ± 13.5 | 35.0 [27.8,44.0] | 45.7 ± 14.7 | 44.0 [35.0,55.0] | 40.8 ± 13.2 | 41.0 [30.5,48.3] | 48.8 ± 19.3 | 44.0 [36.0,59.0] | 48.7 ± 18.3 | 45.0 [36.0,58.0] | 62.0 ± 23.2 | 58.0 [49.8,65.5] | < 0.001 | < 0.001 |
| LA volume, BSA (ml/m^2^) | 21.5 ± 7.4 | 20.4 [16.3,25.7] | 26.3 ± 8.3 | 25.3 [20.2,31.5] | 24.2 ± 8.1 | 25.5 [17.7,27.3] | 23.7 ± 8.6 | 22.0 [18.0,27.8] | 24.2 ± 8.6 | 22.6 [18.4,28.5] | 31.9 ± 11.0 | 30.6 [25.5,33.2] | < 0.001 | 0.853 |
| **Right ventricle** |  |  |  |  |  |  |  |  |  |  |  |  |  |  |
| RV basal diameter (mm) | 31.2 ± 4.1 | 30.8 [28.7,33.6] | 31.2 ± 4.7 | 31.0 [28.7,34.3] | 32.7 ± 4.5 | 32.3 [30.2,34.9] | 37.3 ± 5.1 | 37.0 [34.0,40.5] | 36.3 ± 5.2 | 35.7 [33.3,39.7] | 35.5 ± 6.4 | 35.0 [33.0,38.3] | 0.002 | < 0.001 |
| RV basal diameter, BSA (mm/m^2^) | 18.2 ± 2.3 | 18.3 [16.9,19.7] | 18.1 ± 2.9 | 18.1 [16.2,19.9] | 19.4 ± 2.8 | 19.0 [17.7,21.1] | 18.3 ± 2.6 | 18.0 [16.8,20.3] | 18.2 ± 2.8 | 18.1 [16.3,20.0] | 18.3 ± 3.2 | 18.3 [16.7,20.2] | 0.039 | 0.263 |
| Tricuspid s’ (m/s) | 13.3 ± 2.2 | 13.3 [12.0,15.0] | 12.3 ± 2.3 | 12.0 [11.0,14.0] | 12.8 ± 2.9 | 12.3 [10.5,14.0] | 12.4 ± 2.4 | 13.0 [10.8,14.0] | 12.8 ± 2.6 | 12.7 [11.0,14.2] | 11.7 ± 2.1 | 11.0 [10.5,13.2] | 0.033 | 0.029 |
| TAPSE (mm) | 24.6 ± 3.1 | 24.7 [22.7,26.3] | 24.7 ± 3.2 | 24.3 [22.4,27.3] | 23.1 ± 4.1 | 23.0 [21.0,26.0] | 24.2 ± 3.1 | 24.0 [22.7,25.7] | 24.6 ± 3.8 | 24.3 [21.7,27.7] | 24.0 ± 3.6 | 24.0 [21.3,25.2] | 0.029 | 0.368 |
| **Right atrium** |  |  |  |  |  |  |  |  |  |  |  |  |  |  |
| RA vertical diameter (mm) | 43.0 ± 4.6 | 43.0 [40.8,45.0] | 43.8 ± 4.8 | 44.0 [40.9,46.8] | 43.1 ± 4.7 | 43.0 [40.7,45.3] | 47.7 ± 5.5 | 47.3 [44.7,51.0] | 47.9 ± 4.9 | 48.3 [45.2,51.0] | 50.5 ± 4.8 | 51.3 [47.0,54.1] | < 0.001 | < 0.001 |
| RA horizontal diameter (mm) | 33.9 ± 4.1 | 33.7 [31.3,36.7] | 34.5 ± 4.8 | 34.3 [31.3,37.5] | 34.1 ± 4.1 | 34.3 [31.3,36.7] | 40.2 ± 6.3 | 39.7 [35.3,43.3] | 39.2 ± 5.4 | 39.0 [35.2,42.8] | 38.2 ± 6.4 | 38.0 [35.0,41.7] | 0.247 | < 0.001 |
| RA vertical diameter, BSA (mm/m^2^) | 25.1 ± 2.4 | 25.2 [23.7,26.8] | 25.3 ± 2.8 | 25.4 [23.1,27.0] | 25.6 ± 3.1 | 25.5 [23.8,27.7] | 23.4 ± 2.7 | 23.2 [21.6,25.2] | 23.9 ± 2.4 | 23.9 [22.5,25.5] | 26.1 ± 2.5 | 26.0 [24.6,27.4] | < 0.001 | < 0.001 |
| RA horizontal diameter, BSA (mm/m^2^) | 19.8 ± 2.4 | 19.6 [18.2,21.6] | 20.0 ± 3.0 | 19.7 [18.0,21.6] | 20.3 ± 2.9 | 20.0 [19.0,21.7] | 19.8 ± 3.2 | 19.6 [17.6,21.7] | 19.6 ± 2.8 | 19.7 [17.5,21.7] | 19.7 ± 3.3 | 20.0 [18.2,21.9] | 0.221 | 0.151 |
|  |  |  |  |  |  |  |  |  |  |  |  |  |  |  |
| **Aorta** |  |  |  |  |  |  |  |  |  |  |  |  |  |  |
| Aortic root (mm) | 27.5 ± 2.6 | 27.7 [25.7,29.0] | 29.0 ± 3.4 | 29.0 [26.7,31.3] | 31.3 ± 2.1 | 31.3 [30.0,32.4] | 31.6 ± 3.2 | 31.2 [29.3,33.7] | 33.5 ± 3.7 | 33.3 [31.0,35.6] | 35.8 ± 4.2 | 38.0 [32.8,39.2] | < 0.001 | < 0.001 |
| Aortic root, BSA (mm/m2) | 16.2 ± 1.8 | 16.0 [14.9,17.1] | 16.8 ± 1.9 | 16.9 [15.5,17.8] | 18.1 ± 1.3 | 18.1 [17.2,19.2] | 15.6 ± 1.6 | 15.5 [14.5,16.6] | 16.8 ± 1.7 | 16.8 [15.6,18.0] | 18.4 ± 2.0 | 19.0 [17.3,19.6] | < 0.001 | 0.087 |

Summary values are shown by age and gender subgroups. Due to limited sample size in subgroups and non-normal distributions, parametric range based on ± 1.96 times SD value should be used very cautiously and nonparametric values based on a quantile model from Tables S1-S4 should be preferred. * p-value is based on a quantile regression model estimating median value with age and gender as independent predictors. 25^th^ to 75^th^ percentile is shown along with the median value. BSA, body surface area; LA, left atrium; RA, right atrium; RV, right ventricle; TAPSE, tricuspid annular systolic plane excursion; SD, standard deviation.

**Table S9. Anthropometric variables for healthy population by age and gender.**

| **Gender** | **Females (n = 328)** | | | | | | **Males (n = 247)** | | | | | | **Models*** | |
| --- | --- | --- | --- | --- | --- | --- | --- | --- | --- | --- | --- | --- | --- | --- |
| **Age group** | **Under 40** | | **40-60** | | **Over 60** | | **Under 40** | | **40-60** | | **Over 60** | |  |  |
| **Variable** | **Mean ± SD** | **Median [25^th^,75^th^]** | **Mean ± SD** | **Median [25^th^,75^th^]** | **Mean ± SD** | **Median [25^th^,75^th^]** | **Mean ± SD** | **Median [25^th^,75^th^]** | **Mean ± SD** | **Median [25^th^,75^th^]** | **Mean ± SD** | **Median [25^th^,75^th^]** | **Age**  **p** | **Sex**  **p** |
| **Body surface area**  **(m2)** | 1.7 ± 0.1 | 1.7 [1.6,1.8] | 1.7 ± 0.1 | 1.7 [1.7,1.8] | 1.7 ± 0.1 | 1.7 [1.6,1.8] | 2.0 ± 0.2 | 2.0 [1.9,2.1] | 2.0 ± 0.1 | 2.0 [1.9,2.1] | 1.9 ± 0.1 | 1.9 [1.9,2.0] | 0.025 | < 0.001 |
| **Height (cm)** | 167.8 ± 5.7 | 168.0 [164.0,172.0] | 166.0 ± 6.3 | 165.0 [162.0,170.0] | 162.4 ± 5.3 | 163.0 [158.8,166.6] | 181.2 ± 7.4 | 181.5 [176.0,186.1] | 179.1 ± 6.4 | 179.0 [175.0,183.0] | 174.6 ± 5.9 | 176.0 [171.0,178.0] | < 0.001 | < 0.001 |
| **Weigth (kg)** | 62.9 ± 8.6 | 61.5 [57.0,69.2] | 65.8 ± 8.4 | 66.0 [60.0,71.0] | 65.9 ± 9.0 | 66.2 [57.7,72.2] | 82.9 ± 10.9 | 84.0 [75.3,89.7] | 81.9 ± 10.0 | 81.2 [76.0,89.1] | 78.9 ± 7.9 | 80.4 [74.2,84.8] | < 0.001 | < 0.001 |

* p-value is based on a quantile regression model estimating median value with age and gender as independent predictors. 25^th^ to 75^th^ percentile is shown along with the median value. BSA, body surface area; SD, standard deviation.
